# Paraparesis caused by a cyst in the spinal canal from a pseudarthrosis 22 years following Harrington rod procedure for scoliosis: a case report

**DOI:** 10.1186/1752-1947-6-337

**Published:** 2012-10-03

**Authors:** Adrian Gardner

**Affiliations:** 1 The Royal Orthopaedic Hospital NHS Foundation Trust, Bristol Road South, Northfield, Birmingham, B31 2AP, UK

**Keywords:** Scoliosis, Pseudarthrosis, Harrington rod, Neurological deterioration

## Abstract

**Introduction:**

This case demonstrates very late neurological deterioration due to a pseudarthrosis in the fusion mass after scoliosis surgery. Though not the first case in the literature, it is the first case in which pre-operative magnetic resonance imaging revealed that the compression was due to a cyst arising from the pseudarthrosis.

**Case presentation:**

Twenty-two years after a successful correction and fusion for scoliosis, a 38-year-old Caucasian man presented with progressive numbness and significant weakness. As revealed by imaging, a cyst relating to an old pseudarthrosis was compressing the spinal cord. This was removed, and the cord decompressed, resulting in resolution of all symptoms.

**Conclusions:**

Lifetime care of patients with scoliosis is required for very late complications of surgery. Asymptomatic pseudarthroses have the potential to cause symptoms many years after surgery.

## Introduction

This case report highlights a case of spinal cord compression and clinical neurological deterioration caused by a degenerative cyst arising from a pseudarthrosis in an area of previous scoliosis fusion 22 years after the index surgery. The case highlights the need for long-term follow-up of patients with scoliosis and for patients to have access to scoliosis services to address problems that may arise many years after the original surgery.

## Case presentation

**Figure 1:**
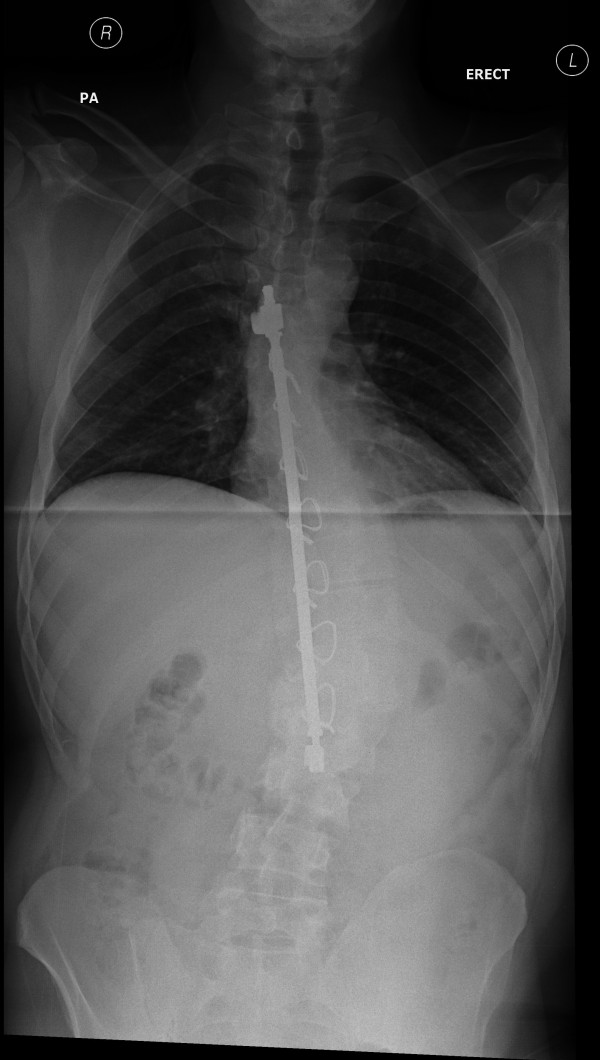
**A pre-operative whole-spine standing radiograph image of the Harrington rod and the scoliosis 22 years after implantation at the index surgery**.

The patient was a Caucasian man who was born in 1974 and who was first referred to the Spinal Deformity Service in 1977. He was initially treated with a brace for his mild scoliosis and followed up over many years until the age of 15, when his scoliosis had progressed, despite bracing, to a 44° curve between T8 and L3. Quite a rapid deterioration had occurred through the adolescent growth spurt, and consequently the patient underwent a posterior Harrington-Luque spinal fusion from T7 to L3. Bone graft was taken from the right iliac crest to create the fusion (Figure 1).

Having had a good result from his surgery, the patient was then discharged at the age of 17 in 1991, and was at that time in full-time employment. Between 1991 and 2012, the patient led a full and active life, worked full-time, played sports (including regular soccer), and was a family man bringing up young children.

In the summer of 2011, the patient, then 38, fell while playing paintball and injured his shoulder. The injury was musculoskeletal and settled reasonably quickly. However, in the two weeks that followed, he started to note increasing left leg weakness that was associated with some reduced sensation. The paraparesis progressively worsened to the point that he was unable to walk without sticks or a wheelchair. There had not been any disturbance in bowel or bladder function. The patient now reported altered sensation down the left side of his body from the L1 dermatome. In a neurological examination of his left leg, his motor power was significantly reduced: he had, at best, 3/5 power but most myotomes were of strength between 1/5 and 2/5 on the Medical Research Council grading scale. There was also blunting of sensation from the L1 dermatome distally.

Spinal radiography at this time showed a good position of the Harrington rod and Luque wires as placed at the index surgery and a reasonable spinal alignment in both planes. Whole-spine magnetic resonance imaging (MRI) revealed a large cervicothoracic syrinx without Arnold-Chiari malformation. This was assumed to be old and not relevant to the current presenting complaint.

**Figure 2:**
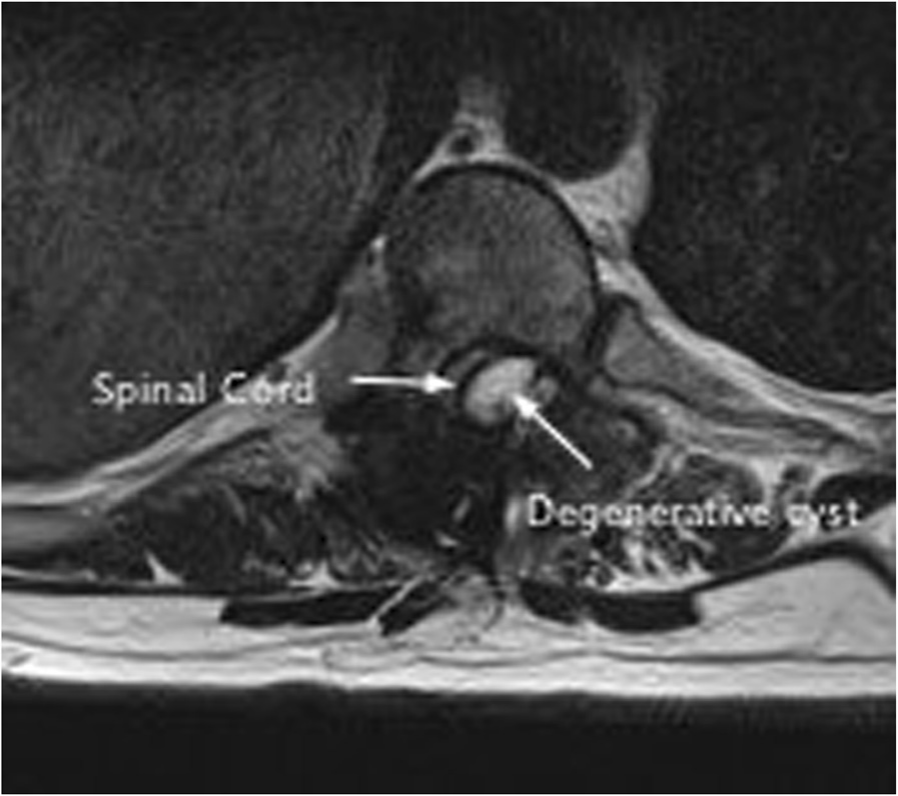
An axial magnetic resonance image of the cyst within the spinal canal and compression of the spinal cord.

**Figure 3:**
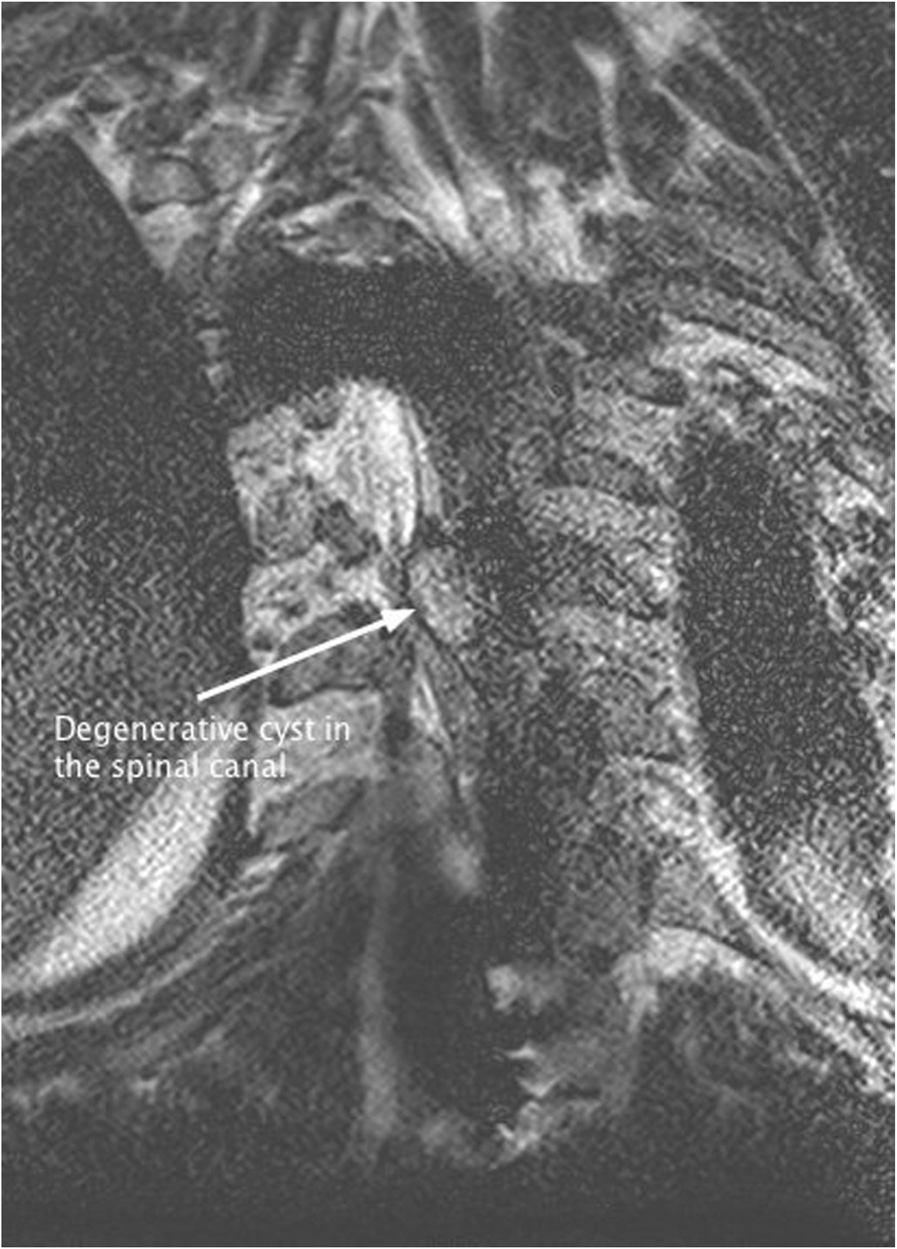
A coronal magnetic resonance image of the location of the cyst relative to the signal void caused by the proximal hook.

**Figure 4:**
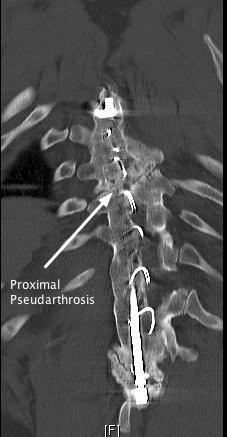
**A coronal computed tomography image of the proximal hook and proximal pseudarthrosis.** When this figure is compared with Figure 3, the pseudarthrosis and cyst are seen to be at the same level within the spine.

The MRI also showed, within the area of instrumentation, a large cyst-like structure causing significant cord compression and this was presumed to be the cause of the recent neurological deterioration (Figures 2 and 3). Computed tomography (CT) of the previously instrumented areas showed two separate pseudarthroses. The most proximal pseudarthrosis corresponded to the level of cord compression as seen on the MRI scan (Figure 4).

The patient underwent urgent surgery to decompress the spinal cord. Instrumentation using a modern pedicle screw system was placed to bridge the level of the proximal pseudarthrosis to provide stability. At the level of the compression on the cord, the fusion mass and pseudarthrosis were then burred away to reveal the spinal canal and cyst, the latter of which was removed and sent for a histological examination. The pseudarthrosis was repaired by using an iliac crest bone graft from the left posterior superior iliac spine (the side opposite that of the index procedure). After surgery, there was a wound breakdown with infection. This was managed through multiple debridements and vacuum-assisted closure therapy followed by a musculocutaneous flap to close the defect with a good result. The histological examination of the cyst revealed a degenerative cyst with no signs of malignancy or infection.

**Figure 5:**
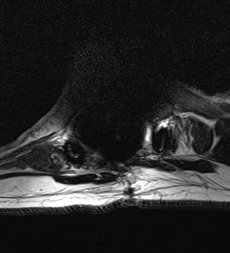
**A post-operative axial magnetic resonance image.** The signal void caused by the presence of the steel and titanium implants obscures any details of the spinal cord or decompression.

**Figure 6:**
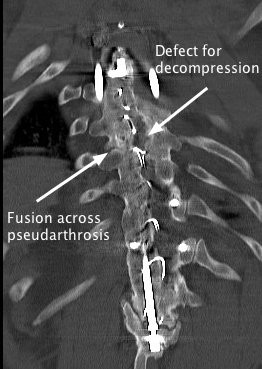
A post-operative coronal computed tomography image of the fusion across the proximal pseudarthrosis and the defect in the fusion mass through which the spinal cord was decompressed.

At last follow-up at six months after surgery, the patient had full recovery of neurological function with no motor weakness or sensory loss and was returning to a more active life. Post-decompression MRI scans are unreadable because of the metal artifact of the Harrington rod and pedicle screw instrumentation over the area in question (Figure 5). A CT scan shows that there is fusion across the upper pseudarthrosis (Figure 6). The lower pseudarthrosis at the level of the distal hook at the Harrington rod is still present but remains asymptomatic at this time. MRI subsequent to the surgery shows no change in the cervicothoracic syrinx seen before surgery.

## Discussion

This patient had, for the time, a well-performed operation with good correction of deformity and a stable construct which allowed him to return to a full and active life for 20 years without problems. It is worth noting that, although there were two separate pseudarthroses in the original fusion mass, both were asymptomatic until a reasonably minor trauma in the summer of 2011. Whether the degenerate cyst from the upper pseudarthrosis would have been present anyway or was stimulated to form after this trauma will never be known. The histology report revealed nothing more than degenerative and inflammatory changes.

Neurological compromise secondary to pseudarthrosis within a previous posterior Harrington rod fusion at some time after the index surgery has been reported previously [1-4]. However, no case is reported with the use of MRI, and the spinal cord compression was demonstrated by using a myelogram at that time. In these cases, the causes of compression were a mix of overgrowth of bone and fibrous tissue. The presence of a degenerative cyst is not noted in any of these cases either on imaging or at surgery. With the advances in imaging in recent years, this case adds to the previous experience another explanation why this late phenomenon may occur and may well have been part of the pathology in the previous documented cases.

The underlying cervicothoracic syrinx is undoubtedly related to why the spinal deformity occurred in the first place but is thought to be unrelated to the acute neurological compromise seen in this case as it was a lower-limb only problem with no symptoms in the upper limbs and the changes seen on the MRI appear to be longstanding.

## Conclusions

This case highlights the need for long-term scoliosis care for patients who have had scoliosis correction, fixation, and fusion in the past. Pseudarthrosis may not be apparent on plain X-ray, and the combination of CT scanning and MRI may be required to make the diagnosis given the difficulties in imaging older implants in an MRI scanner. The degenerative cyst that formed in this case was secondary to the micromovement of the pseudarthrosis, and the location in the spinal canal then caused neurological compromise. Although an operation on an asymptomatic pseudarthrosis may not be appropriate, patients need to be aware of the potential complications of leaving a pseudarthrosis alone given the late complication demonstrated in this case.

## Consent

Written informed consent was obtained from the patient for publication of this case report and accompanying images. A copy of the written consent is available for review by the Editor-in-Chief of this journal.

## Abbreviations

CT: Computed tomography; MRI: Magnetic resonance imaging.

## Competing interests

The author declares that he has no competing interests.
